# Non-suicidal self-injury and emotion regulation: a review on facial emotion recognition and facial mimicry

**DOI:** 10.1186/1753-2000-7-5

**Published:** 2013-02-20

**Authors:** Tina In-Albon, Martina Bürli, Claudia Ruf, Marc Schmid

**Affiliations:** 1 Clinical Child and Adolescent Psychology, Department of Psychology, Universität Koblenz-Landau, Ostbahnstrasse 10, Landau D-76829, Germany; 2 Division of Clinical Psychology and Psychotherapy, University of Basel, Department of Psychology, Basel, Switzerland; 3 Department of Child and Adolescent Psychiatry, University of Basel, Basel, Switzerland

**Keywords:** Non-suicidal self-injury, Emotion regulation, Facial emotion recognition, Facial mimicry, Borderline personality disorder

## Abstract

Non-suicidal self-injury (NSSI) is an increasingly prevalent, clinically significant behavior in adolescents and can be associated with serious consequences for the afflicted person. Emotion regulation is considered its most frequent function. Because the symptoms of NSSI are common and cause impairment, it will be included in Section 3 disorders as a new disorder in the revised *Diagnostic and Statistical Manual of Mental Disorders* (*DSM-5*). So far, research has been conducted mostly with patients with borderline personality disorder (BPD) showing self-injurious behavior. Therefore, for this review the current state of research regarding emotion regulation, NSSI, and BPD in adolescents is presented. In particular, the authors focus on studies on facial emotion recognition and facial mimicry, as social interaction difficulties might be a result of not recognizing emotions in facial expressions and inadequate facial mimicry. Although clinical trials investigating the efficacy of psychological treatments for NSSI among adolescents are lacking, especially those targeting the capacity to cope with emotions, clinical implications of the improvement in implicit and explicit emotion regulation in the treatment of NSSI is discussed. Given the impact of emotion regulation skills on the effectiveness of psychotherapy, neurobiological and psychophysiological outcome variables should be included in clinical trials.

## Introduction

Non-suicidal self-injury (NSSI) is defined as the direct, repetitive, intentional injury of one’s own body tissue, without suicidal intent, that is not socially accepted [1]. The latest studies exploring the occurrence of NSSI in community samples of 14- to 23-year-olds have found its prevalence to range between 10.9 and 38% ([2-5]; see [6] for an overview). However, a one-time occurrence should not be considered pathological. In the diagnostic criteria for NSSI for the fifth edition of the *Diagnostic and Statistical Manual of Mental Disorders* (*DSM-5*) of the American Psychiatric Association [7,8], NSSI needs to occur at least five times to be regarded as problematic. This repetitive NSSI (more than four times) is found in 4–6% of adolescents ([2,5,9]; see [10] for an overview). In a child and adolescent psychiatric inpatient setting, over 25% of adolescents were found to engage in this behavior [2]. Common forms include cutting, severe scratching, burning, and banging or hitting, especially on the arms, legs, stomach, head, and genitals [11]. Rates of NSSI in females and males differ to a much lesser degree than previously assumed [4,12], but females engage in more frequent NSSI than males [13]. The frequency and degree of injuries influence the prognosis of psychotherapeutic treatment [14]. Repetitive NSSI is associated with various concerns, among them depressive symptoms, self-esteem problems, alcohol and drug abuse, interaction problems with peers and family members, poor academic performance, and behavior problems [11,15]. NSSI in adolescence is also a risk factor for NSSI in adulthood and death by suicide [16].

Affective, externalizing, anxiety, substance abuse, and borderline personality disorders are common comorbid diagnoses with NSSI [17]. NSSI most often begins between the ages of 12 and 15 [18] and can last for weeks, months, or even years. It would be erroneous, however, to assume that NSSI is a fleeting adolescent phenomenon. Although the majority of college students surveyed reported stopping within 5 years of starting, it is also clear that the behavior can last well into adulthood [13]. Furthermore, early onset of NSSI is associated with unfavorable treatment outcome in adults [14]. Even though NSSI is a serious condition, only a minority of adolescents receives professional help [2,19].

Because of the frequent occurrence of this serious behavior, the definition of NSSI has become more stringent, and several researchers have proposed that the disorder should be included as a new entity in the revised classification system (*DSM-5*) [7,8], suggesting that NSSI is a common, impairing, and distinctive disorder and therefore should be included in the *DSM* to decrease misperceptions that arise because of a lack of clarity about NSSI’s definition and significance. The list below provides the proposed *DSM-5* criteria for NSSI. NSSI will be included in Section 3 disorders of the *DSM-5*[20], indicating that the criteria set need further research before it will be an official diagnosis. Results of *DSM-5* field trials also suggest further research as two sites had inadequate sample sizes for a successful field trial and for one field trial the estimate of the intraclass kappa was in the unacceptable range [21].

### Diagnostic criteria for non-suicidal self-injury (NSSI) proposed for the fifth edition of the Diagnostic and Statistical Manual of mental disorders^a^

A. In the last year, the individual has, on 5 or more days, engaged in intentional self-inflicted damage to the surface of his or her body, of a sort likely to induce bleeding or bruising or pain (e.g., cutting, burning, stabbing, hitting, excessive rubbing), for purposes not socially sanctioned (e.g., body piercing, tattooing, etc.), but performed with the expectation that the injury will lead to only minor or moderate physical harm. The behavior is not a common one, such as picking at a scab or nail biting.

B. The intentional injury is associated with at least 2 of the following:

1. Psychological Precipitant: Interpersonal difficulties or negative feelings or thoughts, such as depression, anxiety, tension, anger, generalized distress, or self-criticism, occurring in the period immediately prior to the self-injurious act.

1. Urge: Prior to engaging in the act, a period of preoccupation with the intended behavior that is difficult to resist.

1. Preoccupation: Thinking about self injury occurs frequently, even when it is not acted upon.

1. Contingent Response: The activity is engaged in with the expectation that it will relieve an interpersonal difficulty, or negative feeling or cognitive state, or that it will induce a positive feeling state, during the act or shortly afterwards.

C. The behavior or its consequences cause clinically significant distress or interference in interpersonal, academic, or other important areas of functioning. (This criterion is subject to final approval on the use of criteria that relate symptoms to impairment).

D. The behavior does not occur exclusively during states of psychosis, delirium, or intoxication. In individuals with a developmental disorder, the behavior is not part of a pattern of repetitive stereotypies. The behavior cannot be accounted for by another mental or medical disorder (i.e., psychotic disorder, pervasive developmental disorder, mental retardation, Lesch–Nyhan Syndrome, stereotyped movement disorder with self-injury, or trichotillomania).

E. The absence of suicidal intent has either been stated by the patient or can be inferred by repeated engagement in a behavior that the individual knows, or has learnt, is not likely to result in death.

^a^As of November 2012, http://www.dsm5.org.

NSSI is, like suicidal behavior, one of the nine symptoms of borderline personality disorder (BPD) in the *DSM-IV-TR*[22]. BPD is characterized in adolescents and adults by problems with emotion regulation, interpersonal relationships, self-image, affectivity, and impulsivity. However, although NSSI and BPD often co-occur, they also occur independently. It should not be concluded that all adolescents with NSSI fulfill diagnostic criteria for BPD. Even early reports warned against subsuming NSSI under a specific personality disorder. In fact, only about 50% of those who engage in NSSI suffer from BPD [23-25]. Another important distinction has to be made between NSSI and attempted suicide, as the behaviors are indeed different. Three key differences are noteworthy: First, most people engaging in NSSI have no intent to die while conducting the self-injurious act; nevertheless, many people suffering from NSSI report suicide ideas and plans. Second, NSSI is less severe than attempted suicide and usually the damage is not life threatening. Third, NSSI and attempted suicide differ in the frequency of the act, as NSSI often occurs daily [26,27]. In line with several authors (e.g., [28-31]), we propose that NSSI can be regarded as a response for managing or inhibiting aversive emotions, thus representing a dysfunctional emotion regulation strategy.

In the remainder of this consolidated review we provide an overview of emotion regulation and its importance in social relationships. We then focus on two aspects of emotion regulation in adolescents: facial emotion recognition and facial mimicry (the tendency for people to imitate or mimic the facial expressions of others). As mentioned above, interaction problems are often a trigger for NSSI, so the question of what contributes to these social problems should be addressed. Therefore, after we review the empirical studies conducted to date, we attempt to derive suggestions for future theoretical and clinical research.

We have included only published studies with no other limitations. The literature search for this review was conducted in PubMed and PsycINFO using the following keywords: adolescents, non-suicidal self-injury, self-injury, self-harm (but it had to be clear that the self-injury or self-harm was performed without suicidal intent or was not a socially accepted behavior), borderline personality disorder, facial mimicry, and facial recognition. Study selection was independent of date of publication or type of document (review or original research).

### Emotion regulation

**Figure 1:**
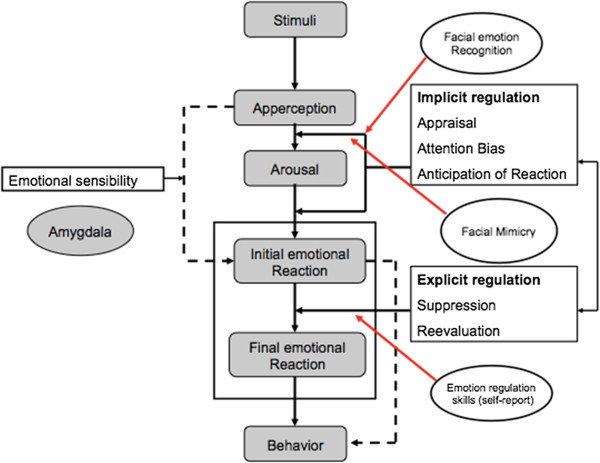
**Herpertz’s affect regulation model**[26]**, including the components facial emotion recognition, facial mimicry, and emotion regulation skills.**

Emotion dysregulation plays a central role in the development and maintenance of mental disorders [32]. Indeed, the majority of disorders in the *DSM-IV-TR* include at least one symptom reflecting a disturbance in emotion regulation [33]. Empirically supported theories of how emotion dysregulation manifests in, maintains, and contributes to mental disorders are increasing, which in turn has stimulated evidence-based treatment development [34]. Figure 1 shows an affect regulation model developed by Herpertz ([35], see also [36]) that describes implicit and explicit affect regulation mechanisms, which can be influenced through arousal, emotional sensibility, and more-or-less helpful and adaptive regulation strategies. Problems in implicit emotion regulation might result from classical conditioning of emotionally stressful experiences to stimuli associated with stressful or traumatic situations. Maladaptive and adaptive explicit emotion regulation strategies might be a result of a person’s social learning history [36].

Increasing attention has been paid to emotion regulation as a potentially unifying function of maladaptive behaviors [37]. The most comprehensive work highlighting emotion dysregulation in psychopathology is Linehan’s [38] biosocial theory on the development of BPD. Her theory has been verified by an increasing number of psychophysiological, genetic, and neuroimaging studies on the development of severe emotion regulation problems, especially in patients suffering from BPD [39,40]. According to this theory, emotion dysregulation is one of the central features of BPD and underlies many associated behaviors of this disorder, including NSSI. This construct of emotion (and thus of emotion dysregulation) is very broad and includes emotion-linked cognitive processes, facial and muscle reactions, action urges, physiology, and emotion-linked actions [39]. Symptoms such as impulsive and NSSI are either the direct or the indirect consequence of emotion dysregulation or attempts to modulate intense emotional reactions [38]. Emotion dysregulation in BPD is hypothesized to consist of greater emotional sensitivity (low threshold for recognition of or response to emotional stimuli) (e.g., [40]), greater emotional reactivity (increased amygdala activity) [41,42], and a slower return to baseline arousal ([43]; for an overview see [44]). Linehan’s conceptualization of NSSI as an emotion regulation strategy is supported by both empirical and theoretical literature on the function of this behavior [45,46]. Many patients with NSSI have major problems with emotion regulation due to biological disposition and an emotionally invalidating environment [38]. An emotionally invalidating environment is one in which a person’s emotional experiences are not responded to in an appropriate or consistent manner. Such an environment does not allow individuals to learn how to regulate intense emotions in an adaptive way and to trust their own experiences as valid and real. Thus, these individuals rely on short-term, impulsive strategies to restore emotions to a tolerable level.

#### Emotion regulation in normal development and in adolescence

Developing skill in emotion regulation involves many factors, including self-awareness of emotion, an appreciation of the origins of emotional experience, an understanding of the potential consequences of emotional expression in different circumstances, and strategies for modifying emotion [47]. The development of emotion regulation begins in early childhood (e.g., sucking a thumb, social referencing) and continues throughout life. In adolescence the understanding of how emotion functions and is managed within oneself becomes evident and provides an important contribution to the emergence of self-understanding [48].

Adolescence is a transition period from childhood to adulthood that is often characterized by instability in body image, identity, and emotion [49]. Key developmental issues in adolescence include autonomy and self-definition, separation from parents, and emotion regulation in physiological and relational maturation. Therefore, adolescence can be considered a period of heightened stress and increased incidence of psychopathology [50]. It is not surprising, then, that pathological personality traits are much more frequent in adolescence than in adulthood [51,52]. Recent neurobiological studies have indicated that structural brain development continues until young adulthood, and neurobiological changes may also impact emotion regulation abilities (see [53-55]). With regard to emotion regulation, there is a change during adolescence in grey and white matter in the cortex [56-58], which could help explain difficulties in cognitive control and emotion regulation during adolescence. Other neurobiological explanations for emotion dysregulation during adolescence are substantial changes in the neurotransmission of dopamine (e.g., [59]) and changes in endocrinology and hormonal status (e.g., [60]). Neurobiological development during adolescence and its influence on the capacity to regulate emotions might be a factor in the peaking of NSSI during adolescence [61].

#### Emotion regulation in the development and maintenance of psychopathology and NSSI

Behavioral theories of psychopathology highlight the importance of the functions that problematic behaviors serve [62]. Emotion regulation is considered the most frequent function of NSSI and is associated with decreases in affective arousal and improvements in affective valence [63-65]. In fact, it is likely that NSSI serves multiple functions simultaneously [66]. The increasing recognition of emotion regulation deficits in NSSI is addressed in the suggested *DSM-5* criteria for NSSI disorder: Criteria B includes emotion regulation deficits—for example, negative feelings or thoughts, such as depression, anxiety, tension, anger, generalized distress, or self-criticism—occurring in the period immediately prior to the self-injurious act; the activity is engaged in with a purpose; this might be relief from a negative feeling/cognitive state or interpersonal difficulty or induction of a positive feeling/state [7]. Emotions such as anger, anxiety, and frustration tend to precede NSSI, which is often followed by feelings of relief and calm in the short term but leads to sadness, guilt, anxiety, disgust, and anger in the long term [65,67,68]. In a physiological study [29], emotional responses to personalized scripts of self-harm incidents in male prisoners with a history of self-injury were examined. Compared to controls, self-harming participants responded with a decrease in physiological arousal and self-reported negative emotion to self-harm imagery but not to the imagery of an accidental injury or a neutral situation. In a second study [69], the tension-reducing effect of self-harming was replicated. Evidence for heightened emotional reactivity and low distress tolerance in adolescents with NSSI was found [70]. In this study adolescents with a history of NSSI reacted with increased skin conductance response to a stress-inducing task and decreased willingness to tolerate this distress and decreased persistence at the task. Several other studies indicated that patients who engage in NSSI are not able to perceive their feelings at all, or sometimes the opposite: that is, they perceive them much too strongly and aversively (e.g., [43,70]). Adolescents with NSSI suffer from intense negative emotions associated with high arousal. Both seem to increase rapidly and can be reduced only by NSSI or other extreme stimuli [70,71]. In addition, adolescents with NSSI have no other way to deal with emotions in social interactions [71].

#### Models of NSSI

To date, there are two theoretical models of NSSI that include an emotion regulation component [72,73]. One is an integrated theoretical model of the development and maintenance of NSSI [73]. The model contains three major propositions. First, NSSI is maintained because it is an effective means of immediately regulating aversive affective/cognitive experiences and/or social situations. Second, the risk of NSSI is increased by distal factors that can lead to interpersonal (e.g., poor communication skills, poor social problem solving) and intrapersonal (e.g., poor distress tolerance, high aversive emotions) vulnerabilities that predispose people to respond to stressful events with affective or social dysregulation, creating a need to use NSSI or other extreme behavior to modulate their experience. Third, the risk of engaging in NSSI is increased by several self-injury-specific factors.

**Figure 2:**
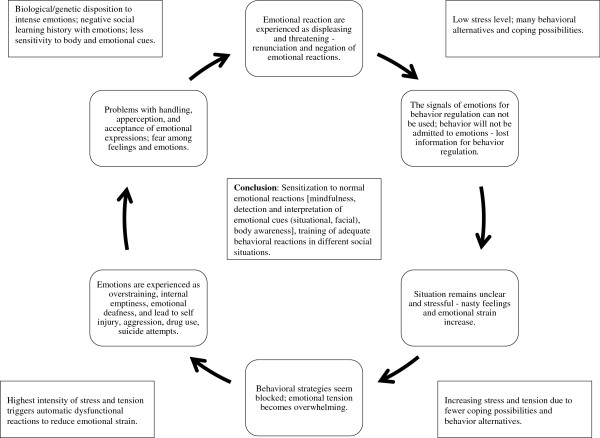
**“Vicious circle” of emotional perception**[62]**.**

The second, experiential avoidance model [72] is an evidence-based theoretical framework highlighting maintenance of NSSI by negative reinforcement of unwanted emotional expressions. Figure 2 shows a “vicious circle,” indicating how difficulties in social interactions may lead to misperceptions and consequently an increase in emotional arousal and a worsening of mood state. This circle highlights one aspect of emotion dysregulation in more detail, whereas the theoretical models [72,73] describe the development and maintenance in a broader way. Our circle concept is meant to be used as a guideline in treatment and psychoeducation. The main aspect of the circle model is that it shows it is necessary to be mindful of the first slight recognitions of emotions and to sensitize patients to their own emotions. The model can help patients understand two things. The initial recognition of emotions is important in choosing adequate behavior strategies and reducing the development of stress and tension. Recognizing an increasing stress level is important for developing skills in stress reduction to prevent self-injury and repeating the experience of the inability to cope with displeasing emotions (see Clinical Implications).

#### Emotion regulation and social relationships

The manner in which people regulate emotions affects their relationships, and vice versa. In social interactions, emotional suppression seems to decrease both negative and positive emotion-expressive behavior, thereby masking important social signals that would otherwise be available to social interaction partners [74]. In addition, the ongoing monitoring of an individual’s own facial expression could distract the suppressing individual and make that person less responsive to the emotional cues of an interaction partner. Studies investigating this theory found that partners of suppressing participants showed greater increases in blood pressure than partners of participants who were either reappraising or acting naturally. These findings indicate that interacting with a partner who shows little positive emotion and who is unresponsive to emotional cues is more physiologically activating than interacting with a partner who shows greater positive emotion and responsiveness [75]. Furthermore individuals who suppressed emotions were less likely to share both their negative and positive emotions with others, had poorer social support, and were less likely to be liked [76]. Recent studies indicate that culture can be a moderator of the effect of emotion suppression on social interactions [77]; further research is needed.

Emotion regulation influences emotional expression and behavior directly. In contrast, the ability to perceive and understand emotions indirectly influences social interaction by helping people interpret internal and social cues, thereby guiding emotional self-regulation and social behavior. Deficits in emotional expression appear to be a risk factor for internalizing and externalizing psychopathology [78]. Emotion perception is an important prerequisite for emotion regulation [79]. If emotional facial expressions are not recognized correctly, emotion regulation will be influenced. Emotion regulation can influence social interaction through several mechanisms. Most saliently, it colors the emotional tone of social encounters. Displays of pleasant emotions tend to elicit favorable responses from others, whereas the expression of negative emotions causes heterogeneous reactions in other people. Most people try to console others who are experiencing negative emotions and offer help and personal support to people who are feeling grief or fear, especially if they are in an emotional relationship with them and doing so might enhance intimacy. This social reinforcement of expressions of negative emotions and moods might have some influence on the development of mental disorders. Some people, however, feel uncomfortable if they are confronted with expressions of negative emotions and drive them away [80].

Chronic difficulties in interpersonal relationships are a core dimension of BPD. BPD patients were found to have fewer social contacts compared to patients with other personality disorders or healthy control groups, and they characterized their social interactions as more disagreeable, ambivalent, angry, empty, and sad [81]. Social situations have been found to be potent triggers for emotional arousal and affective instability in BPD [82]. Interpersonal situations are of high relevance for patients with NSSI. In a German study, adolescents with NSSI (*n* = 220), compared to healthy controls (*n* = 4,693), reported significantly more problems, such as relationship problems within the family and with peers [15]. Interpersonal stress was also associated with engaging in NSSI [83], and quality of peer communication moderated this relationship. Furthermore, adolescents with NSSI often reported engaging in NSSI to influence behaviors of others [83]. It is important to note that a social perspective is compatible with, and meant to supplement, the aforementioned emotion regulation perspective. Patients with NSSI are often not able to tolerate emotional distress and can only regulate subjectively overwhelming and uncontrollable emotions with NSSI. In the modified model (Figure 1), facial emotion recognition and facial emotion expressivity are highly important prerequisites for adequate emotion regulation. In the following, studies investigating facial emotion recognition and facial mimicry are presented in further detail.

### Facial emotion recognition

Facial emotion recognition is impaired in several disorders, such as autism, schizophrenia, depression, anxiety disorders, antisocial personality disorder, and psychopathy. Recognition of facial affect has been investigated mostly using pictures of static or dynamic facial expressions of emotions that have to be attributed to an emotion (see [40] for a review). According to the biosocial theory [38], emotion dysregulation in adult BPD, which is also often characterized by NSSI, is hypothesized to be a consequence of greater emotional sensitivity. In adolescents with NSSI, no studies on facial emotion recognition exist. There are two studies that addressed facial emotion recognition in a sample with adolescents and young adults with symptoms of BPD [84,85]. In the study of Jovev et al. [84], 21 outpatient adolescents meeting three or more *DSM-IV* criteria of BPD and 20 healthy controls participated. They viewed 30 pictures of dynamic facial expressions changing slowly from a neutral face to a prototypical expression of sadness, disgust, surprise, fear, anger, or happiness in 25 steps. The task was to press the space bar as soon as the emotion had been recognized. Image number at the time of response was recorded as an indicator of detection threshold. Recognition accuracy was measured using a forced-choice format, where one of the above-mentioned emotions had to be chosen. Results revealed no heightened sensitivity to emotional expressions in youth with BPD symptoms as compared to healthy controls. Youth with BPD symptoms correctly identified emotional facial expressions at the same threshold of expressivity as healthy controls. The authors suggested that heightened sensitivity to emotional expression might only be apparent in severe BPD or might develop later in the course of the disorder. The second study, investigating facial emotion recognition in female adolescents with BPD, demonstrated that happy facial expressions were perceived as less friendly, and patients perceived them as more threatening than healthy controls did [85]. However, there were no deficits in naming the displayed emotions, nor differences regarding the subjective ratings of the negative and neutral facial expressions.

In summary, the two studies conducted so far indicated that adolescents with symptoms of BPD display a normal ability to recognize facial emotions. The studies used different methods and designs, and certain limitations have to be mentioned. For example, both studies used black-and-white pictures [84,85]. One used a questionnaire to investigate the perception of emotional facial expressions, and static facial expressions [85]. The other had no clinical control group [84]. Due to the low number of studies, results have to be replicated. In addition, the emotional state of the participants should be controlled, for instance, with mood induction.

### Facial mimicry

As is emotion perception and identification, the expression of emotions is important for social interactions. Because humans are a social species, social coordination is essential for survival [86]. Darwin [87] argued that facial expressions of emotion have an adaptive value in social communication because they reveal something about the inner state of the responder that is observable to others. Emotions are highly contagious [80]. An important aspect of emotional contagion, facial mimicry encourages relationships and empathy and therefore represents an important social catalyst [88]. Mirroring emotional facial expressions is a robust effect in healthy persons as they spontaneously and quickly activates congruent facial muscles [89]. Facial mimicry is observable in infants as young as 12–21 days old and plays an important role in establishing attachment through mother–child interactions [90].

Interpersonal situations are highly rule governed and these rules are perceived as normative for interactions [91]. Even minor violations of rules guiding emotional behavior can create substantial problems for the interaction process [86]. Thus, difficulties in social interaction and resulting problems of, for example, social rejection might be understood as violations of social rules due to nonconformity in facial mimicry.

One method used to measure subtle changes in facial muscle activity is electromyography (EMG). EMG analyses are automatic, are more sensitive to subtle muscle activity, and provide exact temporal and quantitative information about the emotional reaction after stimulus presentation [92]. Most research on facial mimicry in children has been conducted in children with autism spectrum disorder (e.g., [93,94]); none has yet been conducted with adolescents with NSSI or BPD. Because of the comorbidity of NSSI and affective disorders [23], we present one study with dysphoric students. The results of this study indicated that in contrast to healthy controls, dysphoric students did not show an increase in *m. zygomaticus* EMG activity in response to happy facial expressions but rather displayed an increase in *m. corrugator* EMG activity [95].

Given the relationship problems of adolescents with NSSI, it might be interesting to explore whether adolescents with NSSI have deficits in facial mimicry, which could lead to problems in social interactions, which trigger NSSI—a vicious circle, perpetuating the problem. Studies investigating these aspects of emotion regulation will provide a better understanding of NSSI and emotion dysregulation. Results will also have theoretical and practical implications for mental health care of adolescents with NSSI.

### Implications

Current research in clinical psychology has increasingly recognized the importance of the assumption that deficits in emotion regulation skills contribute to the development and maintenance of psychopathology (e.g., [96]).

#### Clinical implications

Clinical implications of these studies can be derived from the above-mentioned tasks of emotion regulation. Daily social interactions require a differentiated perception of emotions for adequate contact with fellow human beings. If emotions cannot be identified correctly or are identified relatively late, then this may lead to an increase of emotional arousal and a worsening of mood state, which in a vicious circle may lead to more misperception and finally to strong negative emotions (see Figure 2).

According to Marsha Linehan’s biosocial theory [38] people suffering from NSSI might have problems with emotion regulation because of a biological predisposition in combination with a social learning history of emotional invalidation in their families. These experiences may make them feel uncomfortable with emotions and may lead to a tendency to negate emotions. As a result of this negation of emotions they cannot react adequately to their emotions at a low level of tension. With increasing stress and tension it gets harder and harder to choose appropriate behavior strategies and to solve interactional problems, which could lead to acts of NSSI [44,97].

Affected adolescents may have an increased risk of NSSI, which may be an inadequate attempt to regulate their emotions given the problems they have recognizing their own emotions and interacting with partners’ feelings. If adolescents with NSSI have difficulties in facial emotion recognition and expressions, future treatment and prevention of NSSI would likely benefit from training modules to practice recognizing initial facial expressions in interpersonal situations. Working with video and computer animations may be helpful to support this training process. Because every emotion has a typical physical expression, biofeedback procedures can improve sensitivity to patients’ own emotions. Patients should learn to react immediately to discrete precursors of emotions and should develop the social skills needed to express their emotions adequately and resolve the triggering situation [97]. According to the BPD theory of emotion regulation that is applied to NSSI in adolescents, strong emotion and arousal create the behavioral act of self-injury to modulate negative affects. Therefore, social skills are needed to help prevent and better express heightened emotion. This is important, as evidence on the effectiveness of specific treatments for adolescents with NSSI is lacking. This lack of evidence-based treatments makes treating adolescents with NSSI difficult and often quite scary for clinicians [98,99]. This might explain the high rate of untreated adolescents suffering from NSSI and the high drop-out rates in out- and inpatient psychotherapy [9,92].

Currently, psychotherapies that emphasize emotion regulation, functional assessment, and problem solving appear to be the most effective for treating NSSI [100]. In addition, dialectic behavior therapy (DBT), which is showing preliminary efficacy in adolescents with BPD [99], may be one of the most promising treatments for adolescents with NSSI [98,101]. Linehan et al. [44] suggested it is important in behavior analysis to acknowledge the difference between a high intensity of displayed emotions and a high level of an inappropriate emotion. In the first case, the best intervention will be problem solving, role playing, and the improvement of self-efficacy to cope with difficult interactions. In the second case, it will be necessary for the patient to have the opportunity to reevaluate the emotionally stressful situation and to realize that intense emotion was not necessary and helpful in this situation. To allow reevaluation, an exposure to the emotionally stressful situation will be the best psychotherapeutic intervention. Experiencing that the emotion and interpretations were inadequate in the situation promotes alternative behavior.

To show the effectiveness of skills training to improve implicit and explicit emotion regulation capacities, it might be useful to assess the subjective improvement of patients with evaluated questionnaires. Recent studies indicate that adding psychophysiological and neurobiological measurements may be useful [102,103], nevertheless future research has to show accuracy and feasibility. Successful skills training has been shown to change amygdala activity in response to fearful and disgusting pictures [104]. Other studies have shown different changes in the hypothalamic–pituitary–adrenal axis activity [105].

DBT is a behavioral treatment that draws its principles from behavioral science, dialectic philosophy, and Zen practice. The treatment focuses on factors that maintain dysfunctional behaviors, such as reinforcers of NSSI. Furthermore, DBT emphasizes the balance of acceptance and change [38]. The concept of emotion dysregulation could also be an important topic to address in the psychoeducation of affected family members. Especially for adolescents, it seems very important to do sufficient work with parents, because all family members show a high emotional burden and need help understanding the adolescent and interacting in a way that promotes emotional validation [106,107]. In summary, research in the field of NSSI has increased (especially in relation to BPD), but the number of studies with adolescents still lags far behind the number with adults, despite the prominence of NSSI during this development phase.

#### Theoretical implications

An important methodological aspect of facial affect recognition research is the type of stimuli used. Dynamic facial expressions are more realistic than those that are static and result in stronger activity in the amygdala, a brain area involved in the processing of emotional information [108,109]. Thus, future research on emotion recognition in NSSI should use dynamic facial expressions of emotions. In addition, color pictures of facial expressions might be more realistic than black-and-white images. It will be important to employ a clinical comparison group to ensure the specificity of the results. NSSI is often accompanied by comorbidities [17,23] that are associated with deficits in emotion recognition [40]. Therefore, facial emotion recognition studies in patients with NSSI might profit from large study samples that include comparison groups of individuals with comorbid major depression, anxiety disorders, BPD, and/or other disorders.

Although facial mimicry is a stable effect, studies investigating adolescents with NSSI and BPD should include it as a component, as deficits in facial mimicry can result in social interaction difficulties and misunderstandings and then function as a trigger for NSSI. Studies investigating facial mimicry—such as those on facial recognition—should use dynamic facial expressions because they evoke stronger facial mimicry compared to static expressions [110,111]. Future research directions with clinical implications include intervention studies that examine whether improvement in emotion recognition mediates therapeutic reductions in NSSI and studies of the relation between NSSI and emotion regulation capacity in youths. Results will have theoretical and clinical implications and promote our understanding of the many adolescents suffering from NSSI.

The ethics committee approved the study.

## Competing interest

The authors declare that we have nonfinancial competing interests.

## Authors’ contributions

TI made substantial contributions to the ideas of the paper, the drafting and the revision of the manuscript. MB and CR contributed to the drafting and the revision of the manuscript. MS contributed to the ideas, the drafting and the revision of the manuscript. All authors read and approved the final manuscript.
